# PReS-FINAL-2192: Late cardiac assessment in children who were diagnosed with post streptococcal reactive arthritis - a long term study

**DOI:** 10.1186/1546-0096-11-S2-O27

**Published:** 2013-12-05

**Authors:** Y Uziel, Y Kwint, A Matitiahu, A Levi, L Pearl, J Barash

**Affiliations:** 1 Pediatrics, MEIR HOSPITAL, Kfar Saba, Israel; 2 Pediatrics, Kaplan Medical Center, Rehovot, Israel

## Introduction

In different from rheumatic fever (RF), the association of post streptococcal reactive arthritis (PSRA) and carditis is controversial. Currently the American Heart Association recommends anti-streptococcal prophylaxis for PSRA for one year, repeat echocardiogram and discontinuation of prophylaxis if normal (level 2, C evidence).

## Objectives

To examine whether there is late cardiac involvement in a cohort of children with PSRA.

## Methods

Children with defined PSRA who were followed at the pediatric rheumatology units in Meir and Kaplan hospitals had echocardiography, done by pediatric cardiologists, at least 1 year following diagnosis.

## Results

146 PSRA patients fulfilled the study criteria. Of these, 69 had undergone echocardiography 1-6.9 years from diagnosis. All had normal major parameters. 20 patients had minimal findings: 5 (7.2%) mild mitral insufficiency; 12 (17.4%) minimal mitral insufficiency; 2 (2.9%) mild tricuspid insufficiency and one patient (1.4%) had very mild aortic insufficiency. All were consistent as non significant findings. Of the 77 patients who did not complete echocardiography, 31 were randomly excluded from the study, 26 refused to undergo echocardiography, and 20 were lost to follow-up, all were asymptomatic according to their medical record or telephone questionnaire. There were no statistically significant differences between the group that had completed echocardiography to the group that had not.

## Conclusion

No late cardiac involvement was found in our pediatric PSRA patients. Therefore, different approaches to antibiotic prophylaxis for PSRA and RF are probably justified. A prospective, double blind placebo controlled study is needed to definitively assess the necessity of prophylaxis in PSRA.

## Disclosure of interest

None declared.

